# Remarkably high and accelerating failure rate of a widely used implantable cardioverter-defibrillator lead: A large-scale manufacturer-independent multicenter study with long accurate follow-up

**DOI:** 10.1016/j.hroo.2024.07.010

**Published:** 2024-07-17

**Authors:** Erik F.J. Oosterwerff, Dominic A.M.J. Theuns, Alexander H. Maass, Lieselot van Erven

**Affiliations:** ∗ Department of Cardiology, Isala Klinieken, Zwolle, the Netherlands; † Department of Cardiology, Leiden University Medical Center, Leiden, the Netherlands; ‡ Department of Cardiology, Erasmus University Medical Center, Rotterdam, the Netherlands; § Department of Cardiology, University Medical Center Groningen, Groningen, the Netherlands

**Keywords:** Failure rate, Lead failure, Long-term performance, Linox, Implantable cardioverter-defibrillator, ICD

## Abstract

**Background:**

A high annual failure rate of the Linox family defibrillator lead was reported in various small single-center studies. No independent multicenter long-term performance information exists for this lead.

**Objective:**

Our aim was to assess the longevity of the Linox family leads and to evaluate clinical variables and adverse events associated with failure.

**Methods:**

This 4-center study included adults >18 years of age who received Linox family leads for the prevention of sudden cardiac death. From November 2006 to November 2016, a total of 3993 high-voltage leads of the Linox family were implanted and followed up on.

**Results:**

The absolute failure rate was 10.6% (dwell time to lead failure of 6.3 ± 3.4 years). Multivariate analysis confirmed younger age (for every 5 years younger than 65 years) (hazard ratio 1.09, 95% confidence interval 1.05–1.14, *P* < .001) and subclavian access (hazard ratio 1.46, 95% confidence interval 1.18–1.81, *P* < .001) as independent risk factors for lead failure. Patients frequently presented themselves with inappropriate shocks (20% in patients with lead failure) due to detection of nonphysiologic high-rate signals/noise.

**Conclusion:**

This is the largest physician-driven multicenter study on the very long-term performance of Linox family leads. Our data report a remarkably high failure rate of these leads. Our findings have significant implications for the management of patients. Monitoring by remote care should be available for all active Linox family leads.

**Key Findings**

▪ This is the largest physician-driven multicenter study about performance of the Linox family leads and reports a remarkably high and accelerating failure rate.
▪ The majority of patients with lead failure are presented with nonphysiologic high-rate signals/noise with frequent inappropriate shocks
▪ This study highlights the importance of continuous evaluation of lead performance in real-world populations in particular because early results of the manufacturer’s approval studies reported highly reliable lead performance.

## Introduction

Implantable cardioverter defibrillators (ICDs) are widely used for primary and secondary prevention of sudden cardiac death after several landmark trials have shown the efficacy of these devices.^1^^,^^2^ Generally, ICD systems including electrodes are considered to be safe, with an accepted annual lead failure rate of 0.5%. However, ICD therapy has been associated with a number of short- and long-term complications.^3^, ^4^, ^5^, ^6^ The Achilles heel of a transvenous (ICD) system remains the right ventricular high-voltage lead. In the last 2 decades, 2 ICD leads have been subject to a significant safety issue. The Sprint Fidelis (Medtronic) was prone to fractures and the Riata/Riata ST (Abbott) was found to have insulation failures, with the protective coating degrading over time. These higher-than-expected failures prompted a recall, an increased scrutiny of ICD lead design, and the introduction of new algorithms to postpone inappropriate shocks.^7^^,^^8^

The Biotronik Linox ICD lead family has also been associated with a high failure rate, but this was only made public through a number of small, manufacturer-independent registries.^9^, ^10^, ^11^, ^12^, ^13^, ^14^, ^15^, ^16^ After introduction in 2006 a few changes in lead design were made. Since 2010, the Linox series has been substituted by the LinoxSmart series, covered with an additional SilGlide surface coating, a surface treatment that ensures lubricious coating, improved gliding, and low friction and reduces the risk of abrasion. In a similar manner, Riata ST and Durata (St. Jude Medical) models were provided with an additional abrasion-resistant silicone-polyurethane copolymer (Optim). Silicone rubber is inert and more biostable compared with polyurethane but has a higher coefficient of friction and is more vulnerable to abrasion and breaches.^17^ The second difference concerned the lead body of the DF-1 connector exit, which has been modified to have an increased diameter to strengthen this section. Although there has been concern among device cardiologists because of the occurrence of lead failures in daily practice, no larger-scale studies became available and Biotronik did not issue a field safety notice. For that reason, the Device Advisory Committee of the Netherlands Heart Rhythm Association decided to pool the data from the 4 largest Biotronik ICD implanting centers in the Netherlands in order to determine the true extent of Linox family ICD lead failures, whether predictive factors can be identified, and how failures affect patients.

## Methods

### Study population

The 4 Dutch high-volume ICD implanting centers retrospectively identified all consecutive patients implanted with a high-voltage lead of the Linox family. Data obtained from the review of medical records were demographics (age and sex), lead model, serial number, date of implantation, and implant procedural data. All research activities were conducted according to the principles of the Declaration of Helsinki as revised in 2013. The study was approved by the local Ethics Institutional Committee on Human Research. This was a retrospective cohort analysis of patients with a clinical indication for ICD therapy. The need for written informed consent was waived.

### Description of high-voltage defibrillation leads

**Figure 1:**
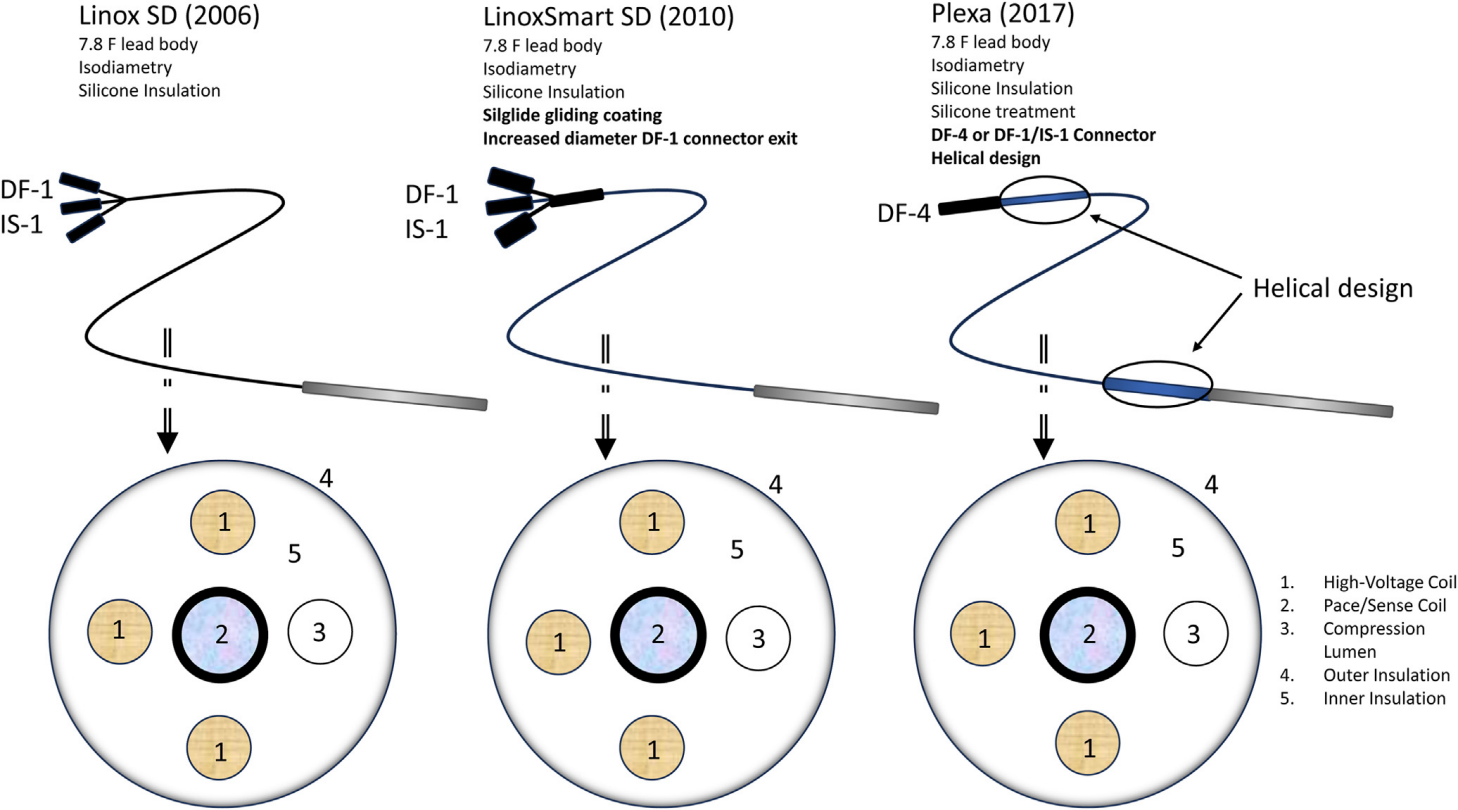
Lead design of the Linox, LinoxSmart, and Plexa.

The Biotronik silicone-insulated Linox defibrillator lead was introduced in 2006. It is a 7.8F single- or dual-coil active or passive fixation, steroid-eluting quadripolar lead with silicone insulation. The pace/sense cable conductor is made of 7 × 7 filars of MP35N R (a nickel-cobalt–based proprietary alloy) material, and the shock coil cable conductor is made of 7 × 7 filars of MP35N R/silver. The pace/sense and shock coil cable conductors are wrapped with a Teflon PFA coating. The inner conductor is a 4 filar wire conductor made of MP35N R. The Linox family has 4 cable lumens to provide a symmetric cross-sectional design. Since 2010, the Linox series has been substituted by the LinoxSmart series. After being approved as magnetic resonance imaging conditional, LinoxSmart was named LinoxSmart ProMRI without any change in its technical design. A more stable lead-to-can connection (DF-4 connector) was developed for the newer generation Biotronik ICD leads, such as the Protego lead, but this was not yet available in the Linox family leads.^17^, ^18^, ^19^ The design of the Protego lead was the basis for Plexa’s lead body design. Plexa and Protego are very similar with isodiametric lead body design and SilGlide. The main differentiator making Plexa one of a kind is the new helical design, which reduces stress on the lead body. Figure 1 presents the different lead designs.

### Definition of lead failure

In the current analysis, lead failure was defined if 1 (or more) of the following criteria were met: (1) presence of nonphysiologic signals on the intracardiac ventricular electrogram, unrelated to external interference; (2) the sudden occurrence pacing impedance outside the interval 200 to 2000 Ω or >100% increase or >50% decrease of the stable baseline impedance; (3) change in high-voltage impedance to >200 or <25 Ω); and (4) a sudden or intermittent increase in right ventricular threshold and/or decrease in R-wave amplitude, without alternative explanation. Lead dislodgment, perforation, T-wave oversensing, R-wave sensing <3.0 mV or >50% reduction, physiological oversensing, gradual increase of impedance, and header problems were not considered as lead failures. Gradually increasing impedance was excluded, as it is not thought to be associated with lead failure, but rather with fibrosis and calcifications at the lead/endocardial surface. The presence of any inappropriate shock associated with lead failure was recorded. All cases associated with lead failure were reviewed and adjudicated by authors E.F.J.O., D.A.M.J.T., L.v.E., and A.H.M. Follow-up started at the time of ICD implantation. In all 4 centers, in-office device interrogation was performed every 6 to 12 months or after symptomatic events. In addition to in-office follow-up, 1323 (33%) patients used remote monitoring of their implanted ICD system.

All patient deaths were recorded. If no lead failure had occurred during follow-up, the date of the last known device interrogation was recorded as the last follow-up date for the Kaplan-Meier analysis. The administrative censoring date was set at June 1, 2022.

### Statistical analysis

Normality of distribution was determined using the Shapiro-Wilk test. Normally distributed continuous variables are expressed as mean ± SD and compared by Student *t* test. Non-normally distributed variables are expressed as median (interquartile range [IQR]) and compared using the Kruskal-Wallis *H* test. Categorical data are expressed as numbers and percentages and compared by the chi-square test or Fisher exact test. Cumulative lead failure rates and cumulative hazards were calculated by the Kaplan-Meier method, and curves were compared by the use of the log-rank test. In addition, the incidence rate of lead failure was calculated using time at risk from implantation to last follow-up. Incidence rates were expressed per 100 patient-years with a 2-sided 95% confidence interval (CI). Comparative analysis was performed by estimating the incidence rate ratio. To assess the change in lead failure risk over time, a log-log plot of the cumulative hazard function (log H vs log t) was constructed. Based on the transition of the cumulative hazard in the log-log plot, conditional survival probabilities of Linox leads functioning normally past the transition point were calculated. Linear regression analysis using the least-squares method was performed to estimate the slope of the conditional survival on the log H vs log t plot. If the slope of the conditional survival log H vs log t approximated 1 (ie, constant lead failure rate over time), linear regression analysis was performed on the cumulative hazard plot (H vs t) to define the lead failure rate per year. Lead failure rates are expressed per 100 patient-years with a 2-sided 95% CI. Patient deaths or lead explantations not related to true lead failure were treated as censoring events. Patients who were lost to follow-up were censored at the time of the last follow-up visit at our clinics. The clinical predictors of lead failure were assessed using a Cox proportional hazards model. Any variable with a *P* value of .2 in a univariate analysis was included in a subsequent multivariate Cox proportional hazards model. Two-sided *P* values of <.05 were considered significant. Statistical analysis was performed using Stata/SE 16.1 for Windows (StataCorp LP) and SPSS version 28 (IBM).

## Results

### Study population

Between November 2006 and November 2016, a total of 3993 high-voltage leads of the Linox family were implanted in 3903 patients. Ninety patients received multiple leads of the Linox family due to infection or dysfunction. A total of 2268 (57%) were the original Linox and 1725 (43%) were LinoxSmart. The various implanted subtypes of the Linox family are summarized in Table 1. Of the study population, 75% (n = 2924) were male, and the mean age of the total population was 65 years (IQR 56–73 years). Most patients had a primary prevention indication (n = 2870 [74%]) and a minority also had a bradycardia pacing indication (n = 810 [21%]). Venous access was obtained through subclavian puncture for most leads (n = 2559 [64%]). In the remaining 1434 (36%) cases, the cephalic approach was used. Patient characteristics at implantation are listed in Table 2. Follow-up was until June 2022, and 1824 (46%) Linox family leads were still functional. Of these functional leads, there were 878 patients with original Linox and 946 with LinoxSmart. The median dwell time was 9.1 years (IQR 7.2–11.1 years), with a significant difference in dwell time between the Linox without Smart and the later introduced LinoxSmart (11.1 years [IQR 6.0–12.3 years] vs 8.4 years [IQR 7.4–9.6 years], *P* < 0.001).

**Table 1:** Subtypes of the different Linox without Smart and LinoxSmart leads.

Subtype Linox family	Number	Fixation	Coils	Insulation
Linox without Smart	2268 (57)	—	—	Silicone
Linox S65	1887 (47)	Active	Single	
Linox SD65	343 (9)	Active	Dual	
Linox S65 Dx	1	Active	Single	
Linox S75	21	Active	Single	
Linox SD75	13	Active	Dual	
Linox T65	2	Passive	Single	
Linox TD65	1	Passive	Dual	
LinoxSmart	1725 (43)	—	—	Silicone with Silglide surface treatment
LinoxSmart S60	3	Active	Single	
LinoxSmart S65	472 (12)	Active	Single	
LinoxSmart S65 Dx	43 (1)	Active	Single	
LinoxSmart SD65	75 (2)	Active	Dual	
LinoxSmart S75	7	Active	Single	
LinoxSmart SD75	2	Active	Dual	
LinoxSmart T65	1	Passive	Single	
LinoxSmart TD65	2	Passive	Dual	
LinoxSmart pro MRI S65	868 (22)	Single	Active	
LinoxSmart pro MRI S65 Dx	229 (6)	Single	Active	
LinoxSmart pro MRI SD 65	13	Dual	Active	
LinoxSmart pro MRI S75	7	Single	Active	

Values are n (%) or n.

**Table 2:** Baseline characteristics (n = 3903).

Age, y	65 (56–73)
Follow-up, y	7.2 (3.3–9.7)
Male	2924 (75)
Primary prevention indication	2878 (74)
Device	
Single chamber	31
Dual chamber	33
CRT	36

Values are median interquartile range) or %.

CRT = cardiac resynchronization therapy.

### Lead failure

**Figure 2:**
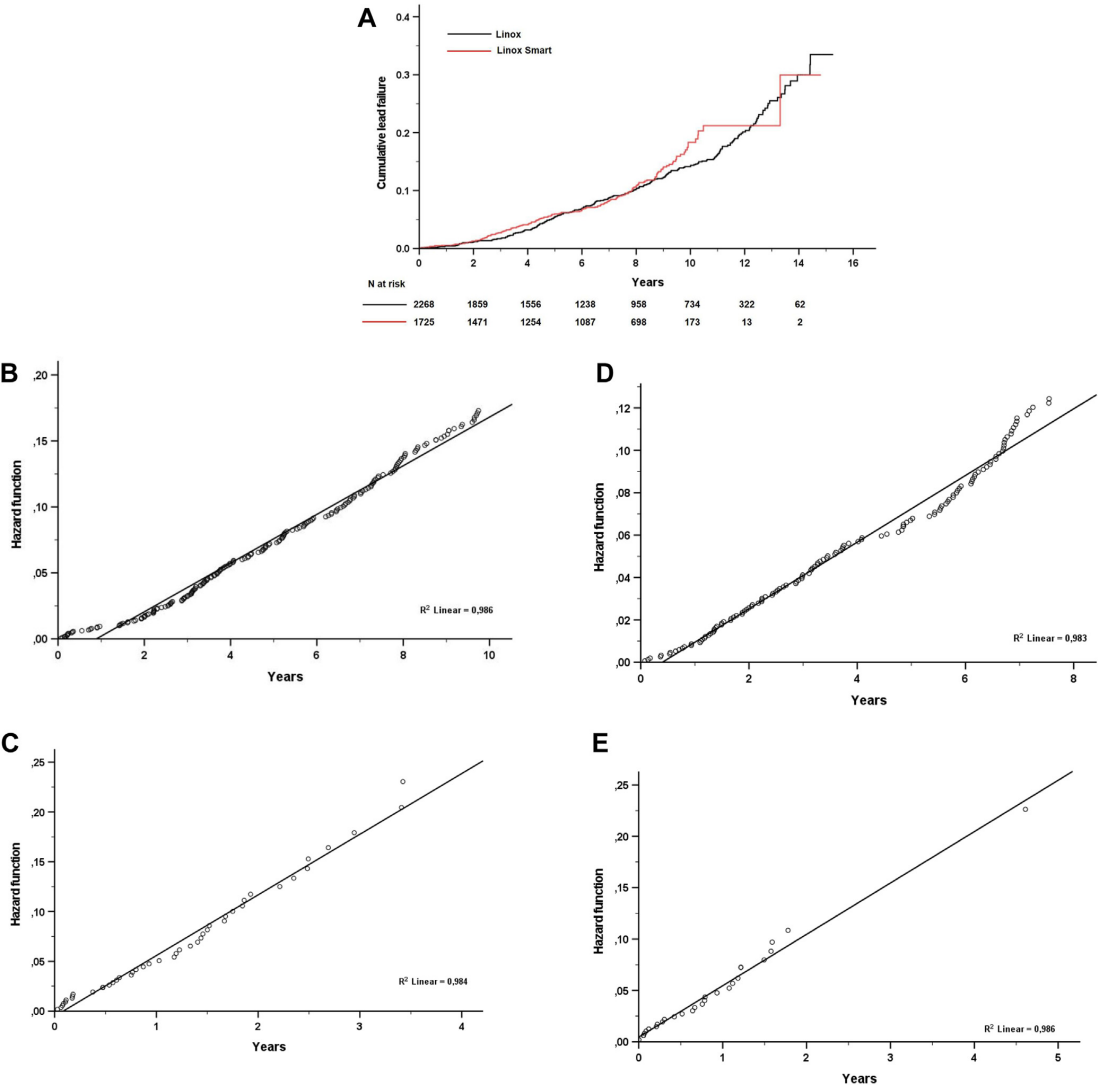
A: Failure rate Linox without Smart and LinoxSmart (*P* = .16). B: Hazard function of conditional survival Linox without Smart after 1.25 years to 11 years (annual failure rate 1.3% per year, 95% confidence interval [CI] 1.3%–1.3%). C: Hazard function of conditional survival Linox without Smart after 11 years (annual failure rate 5.3%, 95% CI 5.1%–5.5%). D: Hazard function of conditional survival LinoxSmart up to 8.7 years (annual failure rate 1.3%, 95% CI 1.2%–1.3%). E: Hazard function of conditional survival LinoxSmart after 8.7 years (annual failure rate 5.1%, 95% CI 4.9%–5.4%).

At June 1, 2022, a total of 422 (10.6%) lead failures had been reported with a dwell time to lead failure of 6.3 ± 3.4 years. There was no difference in absolute failure between Linox without Smart (n = 252 [11.1%]) and LinoxSmart (n =170 [9.9%]) (*P* = .21), but the dwell time to lead failure was significantly shorter with LinoxSmart (5.4 ± 2.9 years vs 6.9 ± 3.6 years, *P* < .001) (Figure 2A).

### Linox without Smart

The cumulative failure rates of the Linox without Smart high-voltage lead were 5.4% and 14.1% at 5- and 10-years’ follow-up, respectively.

Statistical analysis demonstrated a transition of the cumulative failure rate of Linox ICD leads at 11 years’ follow-up. First, all Linox ICD leads up to 11 years’ follow-up were analyzed to assess whether risk of lead failure changed over follow-up to 11 years. Based on the log-log plot, transition of the cumulative hazard occurs at log t = 0.22, which corresponds to a follow-up time of 1.25 years. Based on this, conditional survival for Linox leads functioning normally at 1.25 years up to 11 years was calculated. Linear regression analysis yielded a straight line with perfect fit (R^2^ = 0.97, *P* < 0.0001). Linear regression analysis of the cumulative hazard obtained for functional Linox without Smart leads up to 11 years follow-up yielded a yearly failure rate of 1.3% (95% CI, 1.3%–1.3%, R^2^ = 0.97) (Figure 2B).

For Linox without Smart leads functioning normally after 11 years’ follow-up (n = 556), linear regression analysis yielded a straight line with perfect fit (R^2^ = 0.99, *P* < .0001). For functional Linox leads from 11 years of follow-up onward, a yearly failure rate of 5.3% (95% CI, 5.1%–5.5%, R^2^ = 0.99) was observed (Figure 2C).

### LinoxSmart

The cumulative failure rates of the LinoxSmart high-voltage lead were 6% and 18.3% at 5- and 10-years’ follow-up, respectively. Statistical analysis of this lead demonstrated a transition in the cumulative failure rate at 8.7 years of follow-up. Based on the log-log plot, transition of the cumulative hazard occurred at log t = 0.14, corresponding to a follow-up of 1.15 years. The conditional survival for LinoxSmart leads functioning normally at 1.15 years up to 8.7 years follow-up was calculated. Linear regression analysis of the cumulative hazard obtained for functional LinoxSmart leads up to 8.7 years’ follow-up yielded a yearly failure rate of 1.3% (95% CI, 1.2%–1.3%, R^2^ = 0.99) (Figure 2D).

For LinoxSmart leads functioning normally after 8.7 years’ follow-up, linear regression analysis yielded a straight line with perfect fit (R^2^ = 0.99, *P* < 0.0001). For functional LinoxSmart leads at 8.7 years’ follow-up, the yearly failure rate was 5.1% (95% CI, 4.9%–5.4%, R^2^ = 0.99) (Figure 2E).

### Clinical presentation

**Figure 3:**
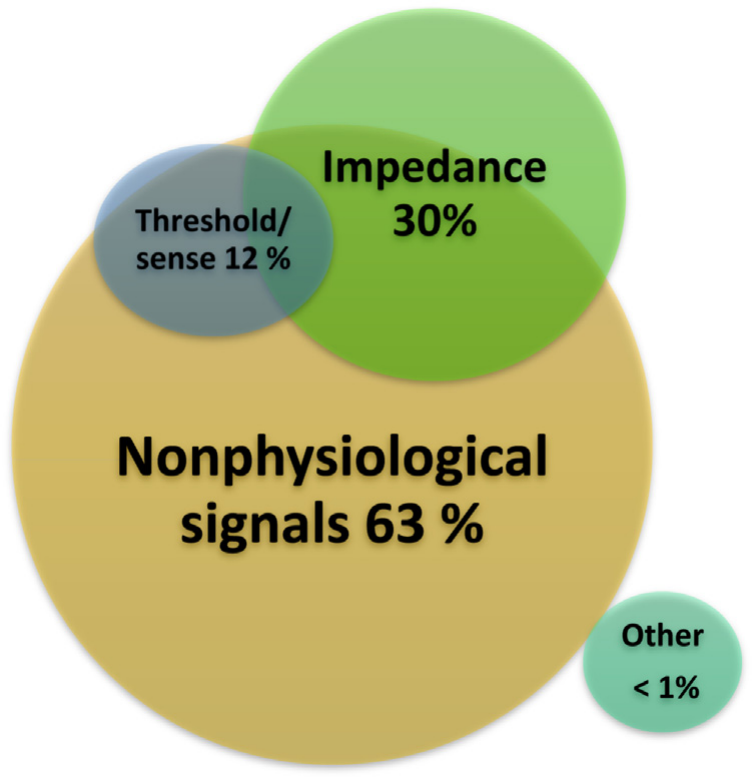
Venn diagram showing lead failure with multiple mechanisms (n = 97 [23%]). In particular, significant threshold/sense changes present with noise and/or impedance changes.

**Figure 4:**
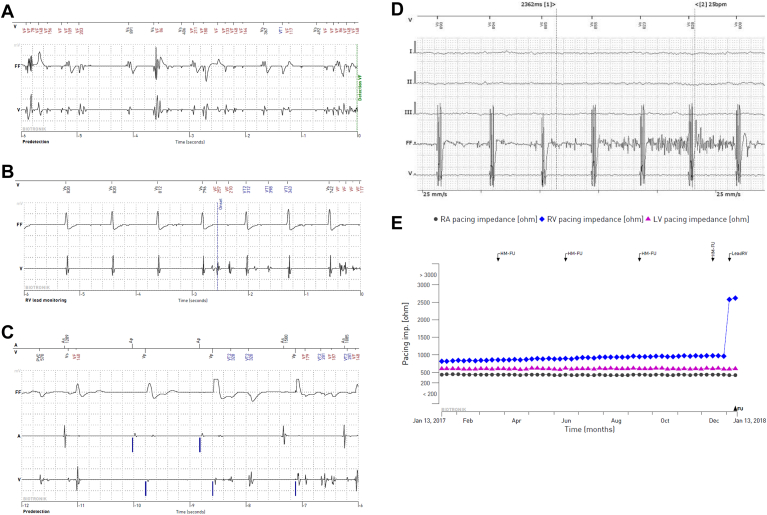
A: Nonphysiologic signals on sensing and shock electrogram (EGM) suggest inner insulation breach. B: Nonphysiologic signals on the sensing EGM. C: Nonphysiologic signals on the sensing EGM and only low-amplitude signals on the shock EGM. D: Nonphysiologic signals on the shock EGM. E: An example of significant changes of lead impedances. Remote care could be effective to detect these lead failures. FU = follow-up; HM = home monitoring; LV = left ventricular; RA = right atrial; RV = right ventricular.

In 325 (77%) of 422 lead failures, there was 1 dominant mechanism. Figure 3 presents the overlapping presentations (23% [n = 97]). Roughly two-thirds (63% [n = 267]) of the patients with lead failure presented with nonphysiologic high-rate signals/noise (see Figures 4A–4D) Most common was nonphysiologic high-rate signals/noise on the near-field channel (90% [n = 240]). Of these patients, 32% (n = 85; 2.1% of the total population) experienced inappropriate shocks. Despite remote care in 1323 patients, 31 patients still experienced inappropriate shocks. Lead failures because of significant changes of lead impedances (see Figure 4E) occurred in 125 (30%) patients. Significant threshold changes happened in 50 (12%) patients. There were no differences in clinical presentation between early and late failures. The distribution of main clinical presentations like oversensing and impedance changes were equal in early and late failures (prevalence of noise Linox failure 62% [<8 years] vs 66% [>8 years], *P* = .3; and prevalence significant impedance change Linox failure 29% [<8 years] vs 31% [>8 years], *P* = .82). The pace-sense component is most prone for failure. Table 3 presents the details. Lead externalization was uncommon (<1% [n = 3]). We studied 49 Biotronik returned product analyses (12% of total failure). Due to the extraction procedures, the leads were damaged and the root causes were not always found. But in 44 cases, it was assumed that the lead had been subject to severe mechanical stress in the implanted state. Interaction between the lead body and the tricuspid valve was mostly taken into consideration as root cause (n = 22 of 44 [50%]) (see Table 3).

**Table 3:** Clinical presentation of a total of 422 lead failures.

Clinical presentation	
Inappropriate shocks	85 (20)
Nonphysiologic high-rate signals/noise	267 (63)
Significant changes of lead impedances	125 (30)
Threshold/sense changes	50 (12)
Lead failure with multiple mechanism	97 (23)
Details nonphysiologic high-rate signals/noise	267
Nonphysiologic high-rate signals/noise near field	240 (90)
Nonphysiologic high-rate signals/noise far field	5 (2)
Nonphysiologic high-rate signals/noise near field and far field	22 (8)
Details significant changes of lead impedances	125
High-voltage component	42 (34)
Pace-sense components	83 (66)
Returned product analysis with considered root cause of failure	44/49 (90)
Severe mechanical stress in the area of the tricuspid valve	22 (50)
Severe mechanical stress due to constant friction against generator	9 (20)
Severe mechanical stress in the area of clavicula-first rib	9 (20)
Severe mechanical stress due to constant friction against a nearby lead	4 (10)

Values are n (%), n, or n/n (%).

### Predictors of lead failure

To identify factors associated with lead failure, we performed univariate and multivariate analysis of baseline characteristics (age, sex, indication), procedure-related variables (venous access and number of leads), and lead-related variables (number of coils). Younger age (for every 5 years younger than 65 years) (HR 1.08; 95% CI 1.04–1.12, *P* < .001) and subclavian access (HR 1.08; 95% CI 1.04–1.12, *P* < .001) were associated with lead failure in this study. Multivariate analysis confirmed younger age (for every 5 years younger than 65 years) (HR 1.09; 95% CI 1.05 to 1.14, *P* < .001), subclavian access (HR 1.46; 95% CI 1.18–1.81, *P* < .001), and single coil (HR 2.15; 95% CI 1.43–3.23, *P* < .001) as independent risk factors for lead failure. Primary prevention was not associated with more lead failure (HR 0.89; 95% CI 0.72–1.10, *P* < .27). A summary is given in Table 4.

**Table 4:** Univariate and multivariate analysis.

	Failure	No failure	Univariate analysis		Multivariate analysis	
			HR (95% CI)	*P* value	HR (95% CI)	*P* value
Age <65 y	265 (64)	1607 (46)	1.08 (1.04–1.12)	<.001	1.09 (1.05–1.13)	<.001
Male	320 (76)	2667 (75)	1.09 (0.88–1.37)	.43		
Primary prevention	299 (71)	2654 (75)	0.89 (0.72–1.10)	.27		
Single coil	397 (94)	3147 (88)	2.15 (1.43–3.23)	<.001	1.45 (1.17–1.80)	<.001
Subclavian access	301 (72)	2258 (64)	1.40 (1.13–1.73)	.002	2.07 (1.37–3.12)	<.001
Single chamber	140 (33)	1077 (30)	Ref			
Dual chamber	142 (34)	1172 (33)	1.04 (0.83–1.32)	.73		
CRT-D	139 (33)	1305 (37)	0.89 (0.70–1.13)	.33		

Values are n (%), unless otherwise indicated.

CI = confidence interval; CRT-D = cardiac resynchronization defibrillator; HR = hazard ratio.

## Discussion

This national, physician-driven, multicenter study on the Biotronik Linox family ICD lead performance in a large real-life population with long accurate follow-up shows a remarkably high absolute lead failure of 10.6% (1.7% per year) and a mean dwell time to lead failure of 6.3 ± 3.4 years, which becomes even higher in the long run. The Linox family ICD lead failure is notably higher in younger patients and with subclavian access. In case of lead failure, 63% presented with nonphysiologic signals and were at risk for inappropriate shock, which actually occurred in about 1:3 of these patients.

The high failure rates observed in this study confirms the results of earlier manufacturer-independent smaller registries^9^, ^10^, ^11^, ^12^, ^13^, ^14^, ^15^, ^16^ but are remarkably higher than the numbers in the last Biotronik product performance report. These showed a 5-year failure rate of only 3.0% (0.6% per year) for the Linox ICD leads.^19^ An explanation for this difference in lead performance could be found in the type of analysis performed by Biotronik and other companies. Their analysis is mostly based on returned product, an inaccurate number because most leads never get returned. Furthermore, Biotronik, like other companies, depends on reporting by physician (eg, battery depletions or lead complications), which leads certainly to underestimations of the true failure rates. This underestimation is further exacerbated since competing risk (ie, mortality) is not taken into account, which means that the number of active leads is much lower than the number of implanted leads. In order to avoid underreporting, postmarketing registries have been performed. The estimated failure rate of the GALAXY (NCT00836589) and CELESTIAL (NCT00810264) registries of Biotronik was 3.7% at 5 years for Linox leads and thus is still much lower than 10.6% at 6.3 years in the current study.^19^ An important difference is the use of remote care. Data collected during remote monitoring visits were not used in the postmarketing registries of Biotronik, while in our registry 33% of the patients were on remote care. Moreover, the current study excels by long duration of follow-up, which was in the Biotronik postmarketing registries only 3.6 years for Linox leads and 2.3 years for LinoxSmart leads. To further analyze the occurrence of lead failure changes over time, we performed a log-log analysis of the cumulative hazard vs time.^20^^,^^21^ This showed that the already high yearly failure rate of 1.3% further increased dramatically after 11 years for Linox to 5.3% and for LinoxSmart even earlier, after 8.7 years, to 5.1%. This shows the importance of very long-term follow-up, which is typically not done by lead registries.

This study highlights the importance of continuous evaluation of lead performance in real-world populations. Postmarketing surveillance of lead performance should be a joint responsibility of patient groups, medical associations, healthcare professionals, medical device manufacturers, and European Union regulators. However, in daily practice, device cardiologists and technicians are dependent on devices companies for quality surveillance. For medical professionals, to recognize a structural problem is already hard, but it is even more difficult to determine how to act, especially because implanted hardware is involved, with the potential complications of surgery being significant and having to be weighed against the risk of unwarranted shocks. Unbiased data from non–industry-related studies of good scientific quality is crucial.^22^^,^^23^

A comparison with the Sprint Fidelis and the Riata/Riata ST lead issues is in order. The yearly failure rates were 4.8% for Sprint Fidelis and 4.9% for Riata/Riata ST leads.^7^^,^^8^ Both leads showed accelerating failure, with a transition already around 4 years for both leads.^20^^,^^21^ The accelerating failure of Linox family leads starts later (around 9 years), but the annual failure rate is even higher and above 5%. The scope of the lead issues with the Linox family leads have similarities to that of the Sprint Fidelis and Riata.^20^^,^^21^^,^^24^, ^25^, ^26^ Furthermore, the number of leads implanted worldwide is similar, estimated between 230,000 and 270,000 worldwide for each.^18^^,^^20^^,^^21^^,^^24^, ^25^, ^26^ It is remarkable that whereas the Sprint Fidelis as well as the Riata failures prompted an FSN and a recall for Medtronic and Abbott, respectively, Biotronik did not come to the same conclusion. This might be related to the underlying mechanisms of the occurring failure, which were multiple in case of the Linox, and therefore difficult to pinpoint. Mechanistically, lead stress due to fraction and movement, as well as abrasion of silicon between the lead and generator, are probably the responsible causes for lead failure of Linox family leads. Insulation damage caused by mechanical stress is time dependent. Therefore, lead failure becomes a more common problem in younger ICD patients and patients with better preserved left ventricular function who have a longer life expectancy. Insulation damage might also depend on the stability of the lead itself and silicone leads therefore also seem to be prone to insulation failure.^6^ In some cases, nonphysiologic high-rate signals/noise were detected on both near- and far-field electrograms (Figure 4A), which is suggestive for inner insulation breach with connection of low- and high-voltage conductors. In 2017, Biotronik introduced a different helical design Plexa lead but has not motivated the significant lead design change. The Linox family leads are no longer commercially available. Currently, an estimated 1800 study patients still have an active Linox family lead with a serious accelerating risk of failure rate. The number of patients worldwide be estimated to be 125,000.

In summary, the Linox family leads have a relatively high failure rate, which becomes even higher in the long run. While the higher-than-expected failures of the Sprint Fidelis and Riata prompted a recall, this was remarkably not the case with the Linox family leads. Patients have experienced the complications related to lead failure, most importantly inappropriate shocks and reoperation, with a severely negative impact on the quality of life.^27^, ^28^, ^29^ Monitoring of electrical parameters by remote monitoring may prevent a subset of inappropriate shocks^30^ and should be available for all active Linox family leads. Moreover, lead integrity alerts and lead noise algorithms could further help to minimalize the clinical impact for the patients. Current-generation Biotronik ICDs are capable of monitoring short intervals as an early sign of lead failure. This has been shown to effectively reduce inappropriate shocks with Sprint Fidelis leads.^29^ The use of an audible or tangible ICD alert function helps monitoring lead integrity by a remote monitor to secure safety. An app on the patient’s mobile phone may replace the monitor in the coming decade, which will surely increase the patient’s safety in case of unreliable hardware.

### Study limitations

Due to the retrospective nature of this study, it may be subject to a reporting bias. The mechanism of lead abnormalities could be established/confirmed in a minority of all leads. In most cases, an additional lead was implanted and the dysfunctional lead was not available for returned product analysis. Gradually increasing impedance was excluded because of the association with fibrosis and calcifications at lead/endocardial surface. We have collected limited baseline patient characteristics and cannot rule out other patient-based factors such as comorbidities affecting lead failure. Considering that structural defects can be electrically silent for an extended period of time, some underreporting of true lead failure may have occurred. This study started in 2006, and at that time remote care was not widely used in the Netherlands.

## Conclusion

This is the largest physician-driven multicenter study about performance of the Linox family leads. Our data report a remarkably high failure rate of the Linox family leads. Besides this, an increase in failure rate during longer follow-up has been shown. The majority of patients with lead failure are presented with nonphysiologic high-rate signals/noise with frequent inappropriate shocks. This study highlights the importance of continuous evaluation of lead performance in real-world populations in particular because early results of manufacturer’s approval studies reported highly reliable lead performance.
